# Effectiveness of a Healthy Lifestyle Program (HeLP) for low back pain: statistical analysis plan for a randomised controlled trial

**DOI:** 10.1186/s13063-021-05591-0

**Published:** 2021-09-22

**Authors:** Emma Robson, Steven J. Kamper, Alix Hall, Hopin Lee, Simon Davidson, Priscilla Viana da Silva, Connor Gleadhill, Christopher M. Williams, for the HeLP Trial Working Group, Damien Smith, Bruce Donald, Catherine Groves, Martin O’Neill, Emma-Leigh Simpson, Kate Reid, Tahila Reynolds, Rebecca Muddle, Lauren Devine, Rebecca Hodder, Amanda Williams, John Wiggers, Karen Gillham, Chris Barnett, Robin Haskins, Andrew Searles, Rod Ling, Erin Nolan, Christopher Oldmeadow

**Affiliations:** 1 School of Medicine and Public Health, University of Newcastle, Callaghan, NSW Australia; 2 Hunter New England Population Health, Wallsend, NSW Australia; 3 School of Health Sciences, University of Sydney, Camperdown, NSW Australia; 4 Nepean Blue Mountains Local Health District, Penrith, NSW Australia; 5 Centre for Statistics in Medicine and Rehabilitation Research in Oxford, Nuffield Department of Orthopaedics Rheumatology and Musculoskeletal Sciences, University of Oxford, Oxford, UK; 6 Hunter Medical Research Institute, New Lambton Heights, NSW Australia; 7 John Hunter Hospital, New Lambton Heights, NSW Australia; 8 NSW Office of Preventative Health, Liverpool, NSW Australia

**Keywords:** Back pain, Lifestyle, Disability

## Abstract

**Background:**

This paper describes the statistical analysis plan for a randomised controlled trial of a Healthy Lifestyle Program (HeLP) for low back pain targeting multiple health risks and behaviours, weight, physical activity, diet and smoking, to improve disability. We describe the methods for the main analyses and economic analysis of the trial.

**Methods and design:**

The trial is a two-arm pragmatic randomised controlled trial comparing the effect of the HeLP intervention to usual care on low back pain disability at 26 weeks. A total of 346 adults with low back pain were recruited from the Newcastle and Hunter region between September 2017 and November 2019 and randomised to either HeLP or usual care. HeLP is a 6-month intervention with participant outcomes measured at weeks 6, 12, 26 and 52 post randomisation. This statistical analysis plan describes data integrity, handling and preparation of data for analyses and methods for analyses. The primary endpoint for the trial is disability at 26 weeks using the 24-item self-report Roland Morris Disability Questionnaire. The primary analysis will follow the intention-to-treat principle using linear mixed regression models.

**Discussion:**

The statistical analysis plan for this trial was produced to reduce outcome reporting bias arising from knowledge of the study findings. Any deviations will be described and justified in the final report.

**Trial registration:**

Australian New Zealand Clinical Trials Registry ACTRN12617001288314. Registered on 6 September 2017.

**Supplementary Information:**

The online version contains supplementary material available at 10.1186/s13063-021-05591-0.

## Background

Addressing lifestyle behaviours in the management of chronic low back pain is recommended but under researched [1]. The Healthy Lifestyle Program (HeLP) for low back pain trial is a randomised controlled trial (RCT) targeting modification of healthy lifestyle and lifestyle behaviours including, excess weight, physical inactivity, poor diet and smoking. Here, we describe a statistical analysis plan to guide data analyses for the trial and outline planned future supplementary analyses.

This statistical analysis plan was written and approved prior to completion of follow-up data collection. All study investigators signed and approved this plan in November 2020. We completed participant enrolment in November 2019, and follow-up data collection will conclude in December 2020. Following data integrity checks, we will lock the database and anticipate that analyses specified in this protocol will be performed between January and September 2021.

## Study overview

### Design

The trial is a two-arm pragmatic randomised trial that investigates the effectiveness of a healthy lifestyle modification program to reduce low back pain disability, compared to usual physiotherapy care. The trial is conducted in the greater Newcastle region of the Hunter New England Local Health District, NSW Australia. The trial was prospectively registered with the Australian New Zealand Clinical Trials Registry (ACTRN12617001288314), and a full protocol has been published elsewhere [2]. The Hunter New England Health Human Research Ethics Committee and University of Newcastle Human Research Ethics Committee approved the trial (Approval No 17/02/15/4.05 and Ref No H-2017-0222 respectively).

Patients with persistent low back pain (*n* = 346) from the greater Newcastle region in NSW, Australia, were referred via general practice, orthopaedic and neurosurgery outpatient clinics or self-referred from community media advertisements. All patients were screened for eligibility via telephone by a trained interviewer. Eligible and consenting patients completed a baseline survey via a computer-assisted telephone interview (CATI) and were then randomised to treatment groups (1:1 ratio).

Participants randomised to the intervention group received HeLP, a 6-month program that included four 45–60-min consultations with a physiotherapist (weeks 1, 3, 6 and 12) and one with a dietitian (week 3), pain education resources (booklet, video, app/website access) and referral to telephone-based health behaviour coaching (the NSW Get Healthy coaching services (GHS) for healthy lifestyle and Quitline for smoking cessation support). Participants randomised to the control group received usual care from an outpatient physiotherapy department in a public hospital. Audit of the physiotherapy department’s usual care prior to the trial showed patients received an average of two clinical appointments. Physiotherapists delivering usual care were asked to not provide lifestyle advice and support over and above what was consistent with their standard care.

*Inclusion/exclusion criteria:*

Adult patients ≥ 18 years of age, who met the following criteria were eligible:

- Primary complaint of chronic low back pain, defined as pain between the 12th rib and buttock crease with or without leg pain for a duration longer than 3 months since onset of pain;
- Average low back pain intensity ≥ 3 out of 10 over the past week, rated on a 0–10 numerical rating scale (NRS), OR at least a moderate level of interference with normal daily activities of daily living (adapted from item 8 on Short Form-36) over the last week;
- Had one or more of the following lifestyle risk factors: overweight ((body mass index (BMI) ≥ 25)), current smoker, lower than recommended physical activity levels (i.e. 30 min of physical activity on at least 5 days of the week); consumes less than 2 serves of fruit and 5 serves of vegetables per day.

Patients were excluded if they:

- Had previously undergone bariatric weight loss surgery;
- Were already undertaking a weight loss or smoking cessation program;
- Had back surgery in the previous 6 months or planned back surgery in the next 6 months;
- Had a known or suspected serious pathology causing back pain (i.e. rheumatoid arthritis, confirmed radiculopathy including both sensory and motor deficits, cancer, fracture or infection);
- Could not actively engage in the intervention (unable to communicate, use a telephone or attend appointments, or unable to adapt meals or exercise);
- Comorbidity that does not allow safe completion of study procedures (e.g. uncontrolled blood pressure or heart conditions, uncontrolled diabetes);
- Could not speak English and refused interpreter services;
- Were pregnant or planning pregnancy in the next 12 months.

Participants who could not speak English were eligible if they accepted the use of interpreter services.

## Objectives

### Primary objective

The primary objective of the trial is to estimate the clinical effectiveness of HeLP on low back pain disability, at 26 weeks, compared to usual care.

### Secondary objectives

Secondary objectives include estimating:

1. The long-term effects of HeLP on low back pain disability at 52 weeks follow-up;
2. The differential effect of HeLP on low back pain disability between overweight/obese and healthy weight individuals at 26 weeks and 52 weeks;
3. The effects of HeLP on secondary outcomes: pain intensity, weight, quality of life (QoL) and smoking behaviour, compared to usual care at 26 weeks and 52 weeks;
4. The effects of HeLP on exploratory outcomes: physical activity, BMI, nutrition quality, sleep quality, pain self-efficacy, psychological distress, alcohol consumption, medication, health care and carer services use, work absenteeism and presenteeism, compared to usual care at 26 weeks and 52 weeks;
5. The cost-effectiveness of HeLP considering health care and societal perspectives.

### Supplementary objectives not included in this SAP

Details of two additional objectives will be reported separately:

1. To evaluate the underling mechanisms of HeLP in a causal mediation analysis for the primary and key secondary outcomes;
2. To complete a mixed methods process evaluation of HeLP.

## Outcomes and data collection

### Data collection and follow-up

Data collection is completed at baseline (prior to randomisation via telephone only) and at 6, 12, 26 and 52 weeks post randomisation. Participants had a choice of completing follow-up data collection, online, via a paper-based form or via telephone with an interviewer (CATI).

Anthropometric data were collected at the initial consultation and week 12 (objective height and weight at the initial consultation, weight only at week 12.)

### Outcome variable definitions

The full list of outcomes and time points appear in Table 1.

**Table 1:** Details of outcome measures and data collection time points

Construct	Measure	Time (week)
**Primary**		
Disability	Roland Morris Disability Questionnaire (RMDQ) [3]	0, 6, 12, 26, 52
**Secondary**		
Pain intensity	11 point (0–10) Numerical Rating Scale [4]	0, 6, 12, 26, 52
Weight	Objective weight measured to the nearest 0.1 kg [5]	1, 12
	Self-reported weight (kg)	0, 6, 12, 26, 52
Quality of life	12-item Short Form Health Survey version 2 (0–100 scale) [6]	0, 6, 12, 26, 52
Smoking status	Current smoker (y/n)^a^ and how many cigarettes smoked per day; from the NSW Population Health Survey [7]	0, 6, 12, 26, 52
**Exploratory**		
Physical activity	International Physical Activity Questionnaire (IPAQ) [8]	0, 6, 12, 26, 52
Body mass index	Calculated using weight (kg) / height (m^2^) (height collected at baseline only)	
Nutrition quality	21-item Food Frequency Questionnaire of intake over the past month [9]	0, 6, 12, 26, 52
Sleep quality	Item-6 from the Pittsburgh Sleep Quality Index^a^ [10]	0, 6, 12, 26, 52
Pain self-efficacy	2-item Pain Self-Efficacy Questionnaire (PSEQ-2) [11].	0, 6, 12, 26, 52
Psychological distress	Kessler 6 Psychological Distress Scale [12]	0, 6, 12, 26, 52
Alcohol consumption	2 items from the Alcohol Use Disorders Identification Test (AUDIT-C) [13]	0, 6, 12, 26, 52
**Economic and process**		
Health care, medication and carer use	Self-reported inventory of health care, community/carer services and medication use for back pain over the last 6 weeks^a^	0, 6, 12, 26, 52
Surgical services		52
Work absenteeism and presenteeism	4 yes/no questions^a^ assessing patient reported appropriateness of, referral, consultation for, or receipt of surgical procedure Self-reported number of days off work due to back pain (absenteeism) and days at work despite feeling ill (presenteeism)	0, 6, 12, 26, 52
Adverse events	Patient reported experience of new medical conditions or an exacerbation of an existing condition^a^ (yes/no, with open text).	6, 12, 26, 52
Satisfaction	Patient reported overall satisfaction with the program using a 0–6 Likert scale (0 not at all satisfied, 6 extremely satisfied)	12, 26

Continuous measures unless indicated ^a^dichotomous

#### Participant demographics and baseline characteristics

Baseline and demographic characteristics will be reported by treatment group. Participant demographics collected only at baseline include age (at the time of randomisation), sex, employment status, income, health insurance status, if back pain is compensable, number of prior episodes of back pain, number of years of pain duration and leg pain involvement. The full list of baseline characteristics and demographics appear in Table 2.

**Table 2:** Demographic and baseline characteristics

Demographic and baseline characteristics	Intervention (***n*** = 173)	Control (***n*** = 173)
Age (years)	Mean (SD)	Mean (SD)
Sex (female)	n/N (%)	n/N (%)
Employment status		
- Employed (full time, part time/casual, self-employed)	n/N (%)	n/N (%)
- Cannot work due to health reasons	n/N (%)	n/N (%)
- Home duties	n/N (%)	n/N (%)
- Student	n/N (%)	n/N (%)
- Retired	n/N (%)	n/N (%)
- Other	n/N (%)	n/N (%)
Income		
- Negative or nil	n/N (%)	n/N (%)
- Up to $33,799 per year	n/N (%)	n/N (%)
- Between $33,800 and $88,399 per year	n/N (%)	n/N (%)
- Between $88,400 and $207,999 per year	n/N (%)	n/N (%)
- ≥$ 208,000 per year	n/N (%)	n/N (%)
- Do not know	n/N (%)	n/N (%)
Private health insurance (y)	n/N (%)	n/N (%)
Back pain duration (years)	Mean (SD)	Mean (SD)
Episodes of back pain which have recovered (number)	Mean (SD)	Mean (SD)
Back pain compensable (y)	n/N (%)	n/N (%)
Back pain with leg involvement (y)	n/N (%)	n/N (%)
Have another co-existing medical condition needing medication (y)	n/N (%)	n/N (%)
Disability RMDQ score (0-24)	Mean (SD)	Mean (SD)
Pain scale score (0–10)	Mean (SD)	Mean (SD)
Self-reported weight (kg)	Mean (SD)	Mean (SD)
Objective measured weight (kg)	Mean (SD)	Mean (SD)
BMI (kg/m^2^)	Mean (SD)	Mean (SD)
Quality of life		
- Physical component score	Mean (SD)	Mean (SD)
- Mental component score	Mean (SD)	Mean (SD)
Smoker (y)	n/N (%)	n/N (%)
> 10 cigarettes per day (smokers only)	n/N (%)	n/N (%)
Physical activity (minutes MVPA/week)	Mean (SD)	Mean (SD)
Nutrition diet quality score (5–15)	Mean (SD)	Mean (SD)
Poor sleep quality	n/N (%)	n/N (%)
Pain self-efficacy (0–12)	Mean, (SD)	Mean, (SD)
Psychological distress (0–24)	Mean, (SD)	Mean, (SD)
Alcohol consumption	Mean, (SD)	Mean, (SD)
Medication use for back pain (y)	n/N (%)	n/N (%)
Additional care for back pain (y)	n/N (%)	n/N (%)
Community or carer support for back pain (y)	n/N (%)	n/N (%)
Number of days off work due to back pain in last 6 weeks	Median (IQR)	Median (IQR)

*y* yes, *RMDQ* Roland Morris Disability Questionnare, *BMI* body mass index, *MVPA* moderate vigorous physical activity, *SD* standard deviation, *IQR* interquartile range

#### Primary outcome

The primary outcome is low back pain related disability measured using the Roland Morris Disability Questionnaire (RMDQ) collected at baseline, weeks 6, 12, 26 and 52 post randomisation [3]. The RMDQ is a self-administered 24-item questionnaire (yes or no responses) that assesses back pain disability related to back pain on the day of questioning. A total scale score (out of 24) is calculated by summing together the number of items for which the respondent answers ‘yes’. Total scores range from 0 (no disability) to 24 (severe disability). The tool has evidence of validity, reliability and sensitivity to change over time and between population groups [3].

#### Key secondary outcomes

To reduce the risk of type 1 error from multiple testing, we prespecified four key secondary outcomes we believed to be of importance for the target group and targets of the intervention. These are collected at baseline, week 6, 12, 26 and 52 defined as:

- *Pain intensity*: Average back pain intensity experienced over the past week, measured on a 0 to 10 continuous NRS, where 0 represents ‘no pain’ and 10 the ‘worst possible pain’ [4].
- *Weight*: measured by self-report in kilogrammes (at all time points) and objectively by treating clinicians (at initial and week 12 consultations only) to the nearest 0.1 kg using International Society for the Advancement of Kinanthropometry (ISAK) procedures [5].
- *Quality of life*: Physical and mental health component scores from the 12-item Short Form Health Survey version 2 (SF12.v2). Continuous score range from 0 to100, with higher scores indicating greater quality of life [6].
- *Smoking status*: Current smoking status (Y/N) and number of cigarettes smoked per day, using 2 items from the NSW Health Survey; ‘Which describes your current smoking status?’ (response options: I smoke daily, I smoke occasionally, I don’t smoke now but I used to, I’ve tried a few times but never smoked regularly, I’ve never smoked) and ‘How many cigarettes smoked per day’ (response options: 1 to 10 cigarettes per day, 11 to 20 cigarettes per day, 21 or more cigarettes per day) [7].

#### Other secondary outcomes

The following secondary outcomes are considered ‘exploratory’ outcomes, collected at baseline, weeks 6, 12, 26 and 52:

- *Physical activity*: International Physical Activity Questionnaire (IPAQ) reported as average hours and minutes spent participating in walking or moderate and vigorous activity [8].
- *BMI*: Calculated as weight (kg)/height (m^2^) using baseline height measurements (see weight outcome).
- *Nutrition*: 21-item Food Frequency Questionnaire of intake over the past month (response options for fruits, vegetables, discretionary choices, whole grains and dairy categories: rarely or never, less than once a week, once a week, 2–3 times a week, 4–6 times a week, 1–2 times a day, 3–4 times a day, 5+ a day, and response options for meat categories: rarely or never, less than once a week, once a week, 2–3 times a week, 4–6 times a week, 7+ times a week); tallied to create a diet quality score (range 5–15) [9].
- *Sleep quality*: Item 6 from the Pittsburgh Sleep Quality Index asking to rate sleep quality in the last week (response options: very bad, fairly bad, fairly good, very good), dichotomised as poor sleep quality (very bad and fairly bad vs. fairly good or very good sleep quality) [10].
- *Pain self-efficacy*: 2 item Pain Self-Efficacy Questionnaire (PSEQ-2) each assessed using a scale of 0–6 with zero indicating ‘not at all confident’ and 6 ‘completely confident’, tallied to create a score out of 12 [11].
- *Psychological distress*: Kessler 6 (K6) Questionnaire; a 6-item scale asking how often a feeling was experienced (nervous, hopeless, restless or fidgety, depressed, everything was an effort, worthless) over the past 30 days (response options: all of the time, most of the time, some of the time, a little of the time, none of the time), tallied to create a score (range 6–30) [12].
- *Alcohol consumption*: 2 items from the Alcohol Use Disorders Identification Test (AUDIT C) tool [13], asking how often alcohol is consumed (response options: never, monthly or less, 2 to 4 times a month, 2 to 3 times a week, 4 or more) and how many standard drinks are consumed on a typical drinking day (response options: refused, 1–2, 3–4, 5–6, 7–9, 10 or more tallied to create a score out of 8.
- *Health care service, medication and community, carer and homecare service use*: self-reported use over the last 6 weeks for back pain; asking ‘Are you currently taking any medications for your back pain?’, ‘Did you use any other health services in the last 6 weeks for your back pain?’, ‘In the last 6 weeks, have other friends or relatives, or community services helped you with tasks at home which you couldn't do because of your back pain?’ (see the ‘Concomitant treatments’ section).
- *Work absenteeism and presenteeism*: Self-reported (number of days) over the last 6 weeks; ‘How many days were taken off normal paid work due to your back pain?’ and ‘How many days were spent at work, despite feeling ill?’

#### Concomitant treatments

Participants are asked to record all medications and health care services (provided outside of the program) used for back pain at baseline and weeks 6, 12, 26 and 52. Medications are recorded as free text, and health care services are selected from a list, or ‘other’, reported as free text. We will code medications using the anatomical therapeutic chemical classification system at the fifth level (Additional file 1 Table 1) [14]. ‘Other’ health will be coded according to common provider types (Additional file 1 Table 2)*.* At 52 weeks, participants are asked specifically if they have seen a surgeon for their back, had back surgery or thought back surgery was still appropriate for their back pain (Additional file 1 Table 2).

#### Economic outcomes

The economic analysis will take the perspectives of the society (including participants) and Australian health system. The time horizon of the economic analysis is 1 year. All costs will be presented in Australian dollars (2019–2020). Outcomes collected to enable economic analyses include:

- *Intervention costs*; calculated from trial records for:
  - Physiotherapist and dietitian consultations based on standard consult costs detailed in the Medical Benefits Schedule (MBS) [15].
  - Payment for labour and materials to design and print written and online resources.
  - GHS and Quitline telephone counselling costs: GHS and Quitline program costs will be estimated using standardised call costs (as provided by the services) and multiplied by the number of calls each participant received. Costs of overheads (e.g. electricity) will be calculated as a percentage of labour costs [16].
- *Healthcare and medication costs*; medication usage and quantity of health care use will be derived from participant surveys, and valuations of these items will use published costs from the websites of the MBS [15] and Pharmaceutical Benefits Scheme (PBS) [17].
- *Community service, carer or homecare costs*: we will use annual carer labour time reported in participant surveys to estimate quantities of relevant hours of community, carer or homecare services. Hours will then be valued according to national average hourly incomes data from the Australian Bureau of Statistics (ABS) [18].
- *Absenteeism and presenteeism costs*; will be estimated using the ‘Human Capital Approach’. Both absenteeism and presenteeism costs will be costed on the basis of the national average daily income as published by the ABS. Absenteeism will be calculated according to participant total reported annual sickness absence due to their low back pain. Presenteeism costs will be calculated by multiplying numbers of annual days reported ‘sick at work’ by productivity reduction factors in the relevant literature [19–21].
- *Quality-adjusted life years*; estimated from the SF-12v2. The data will be transformed to an SF6D format [22, 23].

#### Safety outcomes

Participants were asked if they had any new medical conditions or an exacerbation of another existing condition at all follow-up points. Treating clinicians were asked to report any adverse events (AE) encountered during consultations. We will describe all AEs using the World Health Organization International Classification of Diseases (ICD) codes reported by study group (Additional file 1 Table 3) [24]. Participants are also asked to record any other illnesses requiring medication or health care in the last 2 months from a selected list at baseline and weeks 6, 12, 26 and 52 (Additional file 1 Table 4).

#### Participant satisfaction

All participants rate their satisfaction with overall treatment at weeks 12 and 26 on a 0–6 Likert scale (0 ‘not at all satisfied’, 6 ‘extremely satisfied’).

#### Participant fidelity

We will report the following participant fidelity measures for HeLP (Additional file 1 Table 5):

- The mean number, timing (proportion conducted within 2 weeks of scheduled appointment) and percentage of participants who attended all consultations, collected directly from physiotherapy and dietitian clinic records.
- The number of calls completed, graduations and duration of calls completed with the GHS, collected directly from NSW Get Healthy records.
- The number of calls completed with Quitline (if any) collected via participant self-report at week 52 follow-up.
- The number and percentage of participants who accessed online resources collected from clinic reported records and website analytic data.

We will report physiotherapy attendance in the control group by reporting the mean number and standard deviation (SD) of consultations attended and number and percentage of participants that attended an initial and week 12 consultation.

#### Clinician fidelity

The following measure of the program delivery by clinicians will be reported:

- *Intervention components delivered*; recorded in an intervention checklist which details required components (as prompts) and allows description of any supplementary treatments provided at each consultation. We will report the number and percentage of HeLP intervention components delivered (Additional file 1 Table 6).
- Average duration of appointments.

## Design issues

### Study design

This trial is a two-arm randomised trial (1:1). Consecutive patients referred from primary care, orthopaedic or neurosurgery outpatient clinics and self-referred people seeking care for chronic low back pain were screened for eligibility.

### Treatment allocation

Eligible consenting participants were randomised via a concealed central randomisation service using a prespecified randomisation schedule generated a priori by a statistician using SAS version 9.4. Participants were randomly allocated to HeLP intervention group or control group in a 1:1 ratio using permuted block randomisation, with block sizes of 6 and 4. Randomisation was stratified by BMI category (healthy weight ≥ 18.5–24.9 kg/m^2^, overweight ≥ 25–29.9 kg/m^2^ and obese ≥ 30 kg/m^2^). The randomisation schedule was uploaded into REDCap by the independent statistician. Concealed allocation occurred using the following process: (1) the CATI interviewer, who was not involved in treatment, completed the baseline survey (entered into REDCap) [25] and an automated function in REDCap revealed the participants allocation, and (2) interviewers booked participants into an available initial physiotherapy consultation with either the HeLP or usual care physiotherapists. To minimise performance bias, participants were advised of their consultation details but not informed of their group status.

### Sample size

We used Twisk’s method for mixed models to calculate sample size [26]. We used 4 repeated observations (6, 12, 26 and 52 weeks), an intra-cluster correlation of 0.5, alpha of 5% and allowed for 18% loss to follow-up. Our calculations had two considerations. First, for the primary effect, a sample of 346 participants (173 per group) provides over 90% power to detect a 3-point difference (SD 5) on the RMDQ between intervention and control groups at 26 weeks. This is the considered the Minimal Clinically Important Difference in the primary outcome of disability [27]. We ignored increases in statistical power due to stratification and did not include baseline covariates in the calculation of the sample size. Second, we allowed for a secondary moderation analysis to assess whether the treatment effect varies by BMI classification. A total of 346 participants provides 80% power to detect a differential effect of the intervention in the primary outcome between healthy weight vs. overweight/obese participants (a 2-point difference on RMDQ between the two weight categories, i.e. 5 points in one weight category group vs. 3 points in the other). These estimates are based on baseline prevalence ratio of 20:80 for normal weight and overweight across groups respectively.

## Statistical analysis

### Trial profile

**Fig. 1:**
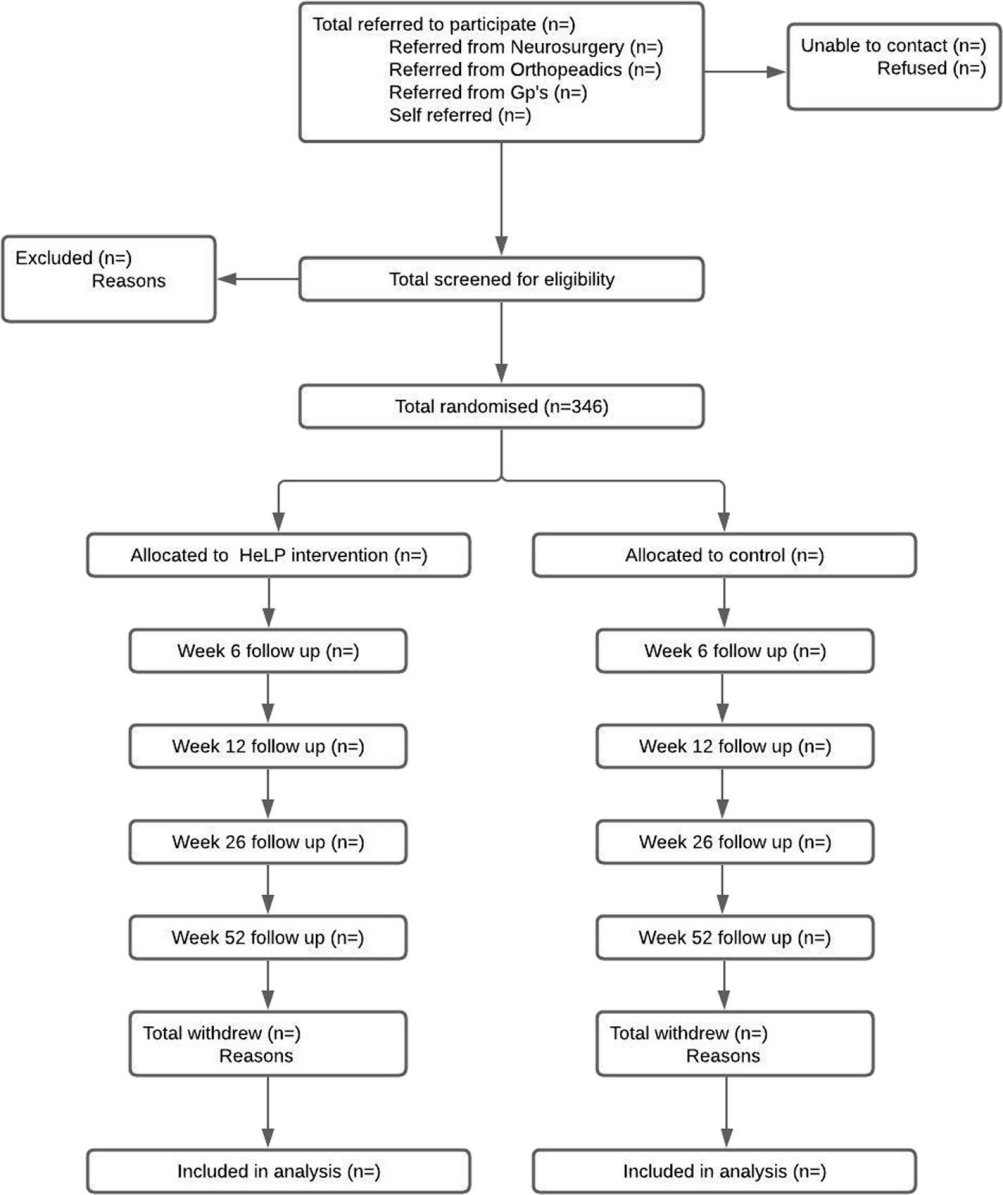
CONSORT diagram

Data will be reported in accordance with the Consolidated Standards of Reporting Trials (CONSORT) guidelines and extension for non-pharmacological interventions [28]. We will report the number of participants who were invited to participate and screened, who met study inclusion criteria or did not and reasons for exclusion, who consented, who were randomised per group and who completed each primary outcome measurement and number of withdrawals with reasons (Fig. 1).

### Data integrity

Data provided by participants is regularly scrutinised for omissions and errors throughout data collection. All manually entered data from paper-based participant surveys will be checked against source records by a second researcher who did not enter data initially. Any inconsistencies will be explored and corrected in consultation with the lead investigator (CW).

As follow-up data can be collected via online, telephone and paper-based surveys, there is possibility of participants answering more than one survey at a time point. In this instance, the order of preference for identifying ‘primary source data’ to be used in the analysis will be based on the following hierarchy:

1. The survey completed by a participant closest to the due time point.
2. If a participant completed two versions of the same survey on the same day, the survey completed directly by the participant was used (i.e. paper or online over telephone interview).
3. When a participant completes two or more survey at one time point, and the primary source (as above) is incomplete, we will supplement any missing items from available secondary sources.

Outliers in the data will be scrutinised by the research team and checked against primary source data. Outliers will be retained if deemed biologically possible otherwise biologically implausible errors that correspond with source data will be considered ‘missing’.

#### Handling decisions for primary outcome data

All efforts are made to confirm data discrepancies with participants. When this is not possible, we will use established methods for handling RMDQ [3]. These include if participants record ‘yes’ and ‘no’ for any item, or indicated that an item was applicable to them (e.g. by writing ‘sometimes’ next to the item), the item is scored ‘yes’; where a participant records the same response for several items (e.g. ‘yes’), and does not mark others, we will consider the missing cells as the alternate response (i.e. ‘no’). As per instructions for the questionnaire, any item not marked is considered not applicable and recorded as a ‘no’ [29].

#### Handling decisions for secondary and exploratory outcomes

We will use standardised protocol instructions for handling data cleaning and scoring of outcomes, where available. These include the *User’s Manual for the SF-12v2® Health Survey* for quality of life data*, Guidelines for data processing and analysis of the IPAQ 2005* [30] for physical activity data and published instructions for scoring pain self-efficacy [11], psychological distress [12, 31] and nutrition data [9]. Alcohol consumption will be calculated manually using criteria from National Health Medical Research Council (NHMRC) guidelines that an individual should not ‘drink more than 10 standard drinks per week’ nor ‘more than 4 standard drinks on any one day’ [32]. All questions requiring ‘open ended’ answers, including medication use, and the type and nature of tasks completed by family, friends or community services will be categorised and coded for analyses by the research team (Additional file 1 Tables 7, 8).

For paper-based participant completed surveys, where discrepancies were recorded such as two answers provided for the one question, participants were contacted to clarify their answers, or a decision was made collectively by the research team.

### General analysis principles

Interim analyses will not be conducted. The primary analysis will be performed by following the intention to treat (ITT) principle whereby all participants will be analysed according to the group they were randomised to, regardless of participant fidelity to treatment or loss to follow-up. Our primary interpretation of the treatment effect will be based on the size and precision of effect estimates using 95% confidence intervals. Significance tests will be used as supporting information for the primary interpretation. All statistical tests will be two-tailed. We will inspect residual plots (residuals vs. fitted, histograms of residuals and random effects plots, to look for evidence of non-constant variance and non-linearity). If there is evidence of assumption departure, we will try different distribution families and link functions (for example ordinal) and present them in a supplementary analysis. Sensitivity analysis will be used to assess the robustness of the primary analysis (see the ‘Sensitivity analyses’ section). Analyses will be conducted using Stata, SAS and/or R by an independent statistician who will be blinded to group status. Variables for group status will be dummy coded to ensure group status remains concealed to the analyst. We will reveal group status after the manuscript is drafted.

We will report the number of observations used in each analysis. Summaries of continuous variables that are normally distributed will be presented as means and SDs or medians and interquartile range for skewed data. Categorical variables will be presented as frequencies and percentages. Percentages will be calculated using the participant count divided by number of participants for whom data is available for as the denominator (for example, nn/NN, %).

### Methods for handling missing data

We will report the number of missing observations for each outcome variable at all time points by treatment group. We will explore patterns of missing data and variables associated with missing data using t-tests for comparing continuous variables and chi-square tests for categorical variables. Reasons for missing data will be reported where known. For the primary analysis, we will also conduct a sensitivity analysis using multiple imputation to handle missing data and present alongside primary analysis results (see the ‘Sensitivity analyses’ section).

### Analysis of baseline characteristics and demographics

Baseline characteristics of included participants will be reported by randomisation group. The full list of baseline characteristics is in Table 2.

### Analysis of the primary outcome

Reporting of the primary, secondary and exploratory outcomes for analyses appears in Table 3.

**Table 3:** Primary, secondary and exploratory outcome analysis at all follow-up time points, week 6, 12, 26 and 52

Outcome Time point	Intervention (***n*** = 172)	Control (***n*** = 172)	Intervention – control^**a**^
**Disability RMDQ score (0–24)**			
Week 6	Mean (SD)	Mean (SD)	Mean diff (95% CI)
Week 12	Mean (SD)	Mean (SD)	Mean diff (95% CI)
Week 26	Mean (SD)	Mean (SD)	Mean diff (95% CI)
Week 52	Mean (SD)	Mean (SD)	Mean diff (95% CI)
**Pain intensity score (0–10)**			
Week 6	Mean (SD)	Mean (SD)	Mean diff (95% CI)
Week 12	Mean (SD)	Mean (SD)	Mean diff (95% CI)
Week 26	Mean (SD)	Mean (SD)	Mean diff (95% CI)
Week 52	Mean (SD)	Mean (SD)	Mean diff (95% CI)
**Weight (subjective, kg)**			
Week 6	Mean (SD)	Mean (SD)	Mean diff (95% CI)
Week 12	Mean (SD)	Mean (SD)	Mean diff (95% CI)
Week 26	Mean (SD)	Mean (SD)	Mean diff (95% CI)
Week 52	Mean (SD)	Mean (SD)	Mean diff (95% CI)
**Weight (objective, kg)**			
Week 12	Mean (SD)	Mean (SD)	Mean diff (95% CI)
**BMI (using subjective height and weight, kg/m**^**2**^**)**			
Week 6	Mean (SD)	Mean (SD)	Mean diff (95% CI)
Week 12	Mean (SD)	Mean (SD)	Mean diff (95% CI)
Week 26	Mean (SD)	Mean (SD)	Mean diff (95% CI)
Week 52	Mean (SD)	Mean (SD)	Mean diff (95% CI)
**Quality of life**			
**Physical component score**			
Week 6	Mean (SD)	Mean (SD)	Mean diff (95% CI)
Week 12	Mean (SD)	Mean (SD)	Mean diff (95% CI)
Week 26	Mean (SD)	Mean (SD)	Mean diff (95% CI)
Week 52	Mean (SD)	Mean (SD)	Mean diff (95% CI)
**Mental component score**			
Week 6	Mean (SD)	Mean (SD)	Mean diff (95% CI)
Week 12	Mean (SD)	Mean (SD)	Mean diff (95% CI)
Week 26	Mean (SD)	Mean (SD)	Mean diff (95% CI)
Week 52	Mean (SD)	Mean (SD)	Mean diff (95% CI)
**Smoker (y)**			
Week 6	n/N (%)	n/N (%)	OR (95% CI)
Week 12	n/N (%)	n/N (%)	OR (95% CI)
Week 26	n/N (%)	n/N (%)	OR (95% CI)
Week 52	n/N (%)	n/N (%)	OR (95% CI)
**Physical activity**			
**Minutes/week of at least moderate activity**			
Week 6	Mean (SD)	Mean (SD)	Mean diff (95% CI)
Week 12	Mean (SD)	Mean (SD)	Mean diff (95% CI)
Week 26	Mean (SD)	Mean (SD)	Mean diff (95% CI)
Week 52	Mean (SD)	Mean (SD)	Mean diff (95% CI)
**MET-minutes/week of low physical activity levels**			
Week 6	Mean (SD)	Mean (SD)	Mean diff (95% CI)
Week 12	Mean (SD)	Mean (SD)	Mean diff (95% CI)
Week 26	Mean (SD)	Mean (SD)	Mean diff (95% CI)
Week 52	Mean (SD)	Mean (SD)	Mean diff (95% CI)
**MET-minutes/week of moderate physical activity levels**			
Week 6	Mean (SD)	Mean (SD)	Mean diff (95% CI)
Week 12	Mean (SD)	Mean (SD)	Mean diff (95% CI)
Week 26	Mean (SD)	Mean (SD)	Mean diff (95% CI)
Week 52	Mean (SD)	Mean (SD)	Mean diff (95% CI)
**MET-minutes/week of high physical activity levels**			
Week 6	Mean (SD)	Mean (SD)	Mean diff (95% CI)
Week 12	Mean (SD)	Mean (SD)	Mean diff (95% CI)
Week 26	Mean (SD)	Mean (SD)	Mean diff (95% CI)
Week 52	Mean (SD)	Mean (SD)	Mean diff (95% CI)
**Nutrition diet quality score (5–15)**			
Week 6	Mean (SD)	Mean (SD)	Mean diff (95% CI)
Week 12	Mean (SD)	Mean (SD)	Mean diff (95% CI)
Week 26	Mean (SD)	Mean (SD)	Mean diff (95% CI)
Week 52	Mean (SD)	Mean (SD)	Mean diff (95% CI)
**Poor sleep quality (y)**			
Week 6	n/N (%)	n/N (%)	OR (95% CI)
Week 12	n/N (%)	n/N (%)	OR (95% CI)
Week 26	n/N (%)	n/N (%)	OR (95% CI)
Week 52	n/N (%)	n/N (%)	OR (95% CI)
**Pain self-efficacy (0–12)**			
Week 6	Mean (SD)	Mean (SD)	Mean diff (95% CI)
Week 12	Mean (SD)	Mean (SD)	Mean diff (95% CI)
Week 26	Mean (SD)	Mean (SD)	Mean diff (95% CI)
Week 52	Mean (SD)	Mean (SD)	Mean diff (95% CI)
**Psychological distress (6–30)**			
Week 6	Mean (SD)	Mean (SD)	Mean diff (95% CI)
Week 12	Mean (SD)	Mean (SD)	Mean diff (95% CI)
Week 26	Mean (SD)	Mean (SD)	Mean diff (95% CI)
Week 52	Mean (SD)	Mean (SD)	Mean diff (95% CI)
**Usual alcohol consumption (0–8)**			
Week 6	Mean (SD)	Mean (SD)	Mean diff (95% CI)
Week 12	Mean (SD)	Mean (SD)	Mean diff (95% CI)
Week 26	Mean (SD)	Mean (SD)	Mean diff (95% CI)
Week 52	Mean (SD)	Mean (SD)	Mean diff (95% CI)
**Medication use for back pain in previous 6 weeks (y)**			
Week 6	n/N (%)	n/N (%)	OR (95% CI)
Week 12	n/N (%)	n/N (%)	OR (95% CI)
Week 26	n/N (%)	n/N (%)	OR (95% CI)
Week 52	n/N (%)	n/N (%)	OR (95% CI)
**Use of other health care services for back pain in previous 6 weeks (y)**			
Baseline	n/N (%)	n/N (%)	OR (95% CI)
Week 6	n/N (%)	n/N (%)	OR (95% CI)
Week 12	n/N (%)	n/N (%)	OR (95% CI)
Week 26	n/N (%)	n/N (%)	OR (95% CI)
Week 52	n/N (%)	n/N (%)	OR (95% CI)
**Carer, community or homecare services use in previous 6 weeks (y)**			
Week 6	n/N (%)	n/N (%)	OR (95% CI)
Week 12	n/N (%)	n/N (%)	OR (95% CI)
Week 26	n/N (%)	n/N (%)	OR (95% CI)
Week 52	n/N (%)	n/N (%)	OR (95% CI)
**Absence from work in last 6 weeks due to back pain (days)**			
Week 6	Mean (SD)	Mean (SD)	Mean diff (95% CI)
Week 12	Mean (SD)	Mean (SD)	Mean diff (95% CI)
Week 26	Mean (SD)	Mean (SD)	Mean diff (95% CI)
Week 52	Mean (SD)	Mean (SD)	Mean diff (95% CI)
**Days at work ill with back pain in last 6 weeks (number)**			
Week 6	Mean (SD)	Mean (SD)	Mean diff (95% CI)
Week 12	Mean (SD)	Mean (SD)	Mean diff (95% CI)
Week 26	Mean (SD)	Mean (SD)	Mean diff (95% CI)
Week 52	Mean (SD)	Mean (SD)	Mean diff (95% CI)
**Adverse events (y)**			
Week 6	n/N (%)	n/N (%)	OR (95% CI)
Week 12	n/N (%)	n/N (%)	OR (95% CI)
Week 26	n/N (%)	n/N (%)	OR (95% CI)
Week 52	n/N (%)	n/N (%)	OR (95% CI)

^a^Adjusted as per analysis approach. *RMDQ* Roland Morris Disability Questionnare, *BMI* body mass index, *y* yes, *Mean diff *mean difference between groups, *CI* Confidence interval, *SD* standard deviation, *OR* odds ratio

Mixed models for repeated measures will be used to estimate the effectiveness of HeLP (compared to usual care), for back pain disability (RMDQ), using data from all randomised participants at week 6, week 12 and week 26 time points. The model will include fixed effects for time, group, the baseline value of the outcome, stratification variable (BMI category), a time by group interaction term and an unstructured covariance matrix to model the within-participant error. The time by group interaction will be used to assess the between-group differences in mean disability scores at any time point and will remain in the model regardless of its *p* value. The primary analysis will not adjust for prognostic covariates; however, a covariate adjusted model will be presented as a sensitivity analysis (see the ‘Sensitivity analyses’ section). We will report the least square mean difference at each time point and associated 95% confidence intervals. We will use the Kenward-Roger method of denominator degrees of freedom estimation.

Between-group comparisons at 26 weeks (aligning with completion of the intervention) will be considered the primary endpoint of the trial. Between-group comparisons at 52 weeks will be secondary end-points, assessed to consider longer term effects.

### Moderation analysis

We will conduct a moderation analyses to assess whether the treatment effect (primary endpoint at 26 weeks) varies by baseline BMI classification. This will include a three-way interaction term of time by group by baseline BMI (dichotomised; healthy weight ≥ 18.5–24.9 kg/m^2^, overweight ≥ 25) and all relevant lower order terms from the primary model.

### Analysis of secondary outcomes

We will test four pre-specified ‘key’ secondary outcomes (pain, weight, quality of life, smoking). Other secondary outcomes will be analysed as exploratory outcomes and include, physical activity, nutrition, BMI, sleep, psychological distress, pain self-efficacy, alcohol consumption, health care use, medication use, carer or community service use, other illnesses, work absenteeism and presenteeism. All secondary outcomes will be analysed using mixed models for repeated measures for continuous outcomes and logistic mixed-effects regression models for dichotomous outcomes, using data for all randomised patients at all time points (weeks 6, 12, 26 and 52). The fixed effects specified for the main outcome model will be included in the analysis of secondary and exploratory outcomes. We will report the between-group mean differences for continuous measures and consider appropriate measures of association [33] (relative and absolute) for binary outcomes, along with 95% confidence intervals. Secondary outcome analyses will not be adjusted for prognostic variables.

### Sensitivity analyses

CACE analyses will be conducted for the primary and key secondary outcomes (pain, weight, quality of life, smoking) to investigate treatment effects amongst compliers at 26 week and 52 week endpoints. Based on consensus within the investigator group, compliance will be attendance of at least two intervention consultations plus five or more GHS telephone calls (or earlier agreed graduation as per GHS protocol). For CACE analyses, we will assume allocation to treatment group does not affect outcomes of participants who do not comply with treatment. We will use instrumental variable (IV) regression to assess the causal effect of HeLP treatment on the primary and secondary outcomes in compliers [34]. IV regression will be carried out using the two-stage least squares estimator (command ‘ivregress 2sls’) in Stata.

We will conduct a sensitivity analysis of the primary analysis adjusting for prognostic variables that are unbalanced between groups at baseline. This analysis will be conducted if there is imbalance in any of the following potentially prognostic variables at baseline: back pain duration, disability, pain intensity, BMI, back pain compensation, age, smoking, psychological distress and pain self-efficacy. Imbalance is defined as the difference between groups being > 20% of the inferior group score. Imbalanced variables will be added as fixed effects in the primary analysis model. A final sensitivity analysis of the primary analysis will be conducted using multiple imputation. For imputation, we will use baseline demographics, other non-missing RMDQ scores and treatment group. We will increase the number of iterations used until the resulting imputations do not change widely between runs and use 20 burn ins.

### Analysis of adverse events

Fisher’s exact test will be used to compare the incidence of AEs between groups. This test will be used as we expect the rate of AEs will be low.

### Economic analyses

Cost collection for economic analyses has been described in the ‘*Economic outcomes*’ section. Three economic analyses will be conducted. First, we will conduct a costing study comparing the costs of the HeLP intervention and usual care control groups by calculating mean costs between groups required to deliver each treatment. Second, we will conduct a cost utility analysis from societal perspective which will include costs of lost participant productivity (absenteeism and presenteeism) and non-health care costs such as unpaid carer and support services. The sample size will be the same as for the primary clinical measure of effect and costs will be assigned to participants on an intention to treat (ITT) basis. Third, we will conduct a budget impact analysis (BIA) of comparing the affordability of the intervention and usual care from the healthcare perspective.

For the cost utility analysis, health state utilities transformed from SF-12 score via the SF-6D algorithm will be used to estimate quality-adjusted life years (QALYs) and combined with societal level costs over 52 weeks. We will calculate an incremental cost utility ratio, which will compare the ratio of the differences in costs to differences in QALYs.

### Supplementary analyses

Details and results of the following two supplementary analyses will be reported separately.

A causal mediation analysis will be conducted to investigate treatment mechanisms using data collected at baseline, 12 and 26 weeks. If the HeLP intervention is found to be effective, we will estimate the extent to which pre-determined mediators explain the treatment effect. If not effective, we will identify where the hypothesised causal pathway broke down. We will estimate the mediating effects of weight, physical activity, diet, smoking, pain self-efficacy, and psychological distress on low back pain disability (RMDQ), pain intensity (NRS), and quality of life (SF12V2). We will also investigate the extent to which concomitant interventions explain the effect of group allocation on outcomes. We will adjust for the following confounders of the mediator-outcome effect: baseline pain duration, weight, disability, pain and QoL.

A mixed methods process evaluation of the HeLP intervention will be conducted using quantitative data collected throughout the trial follow-up and qualitative data collected after 52 weeks follow-up. Quantitative data includes administrative data recorded by treating intervention clinicians and participants’ self-reported data collected during follow-up. Qualitative data was collected using semi-structured interviews or focus groups with a purposeful sample of participants and all clinicians at completion of the study. Using quantitative and qualitative data, we will assess and describe the extent to which the intervention was delivered according to protocol (*fidelity*), participant adoption of the components of the HeLP intervention (*adoption*), explore and describe if HeLP was perceived as agreeable/satisfactory (*acceptability*), perceived to be an appropriate fit for treating back pain (*appropriateness*) and the extent HeLP can be carried out in a healthcare setting (*feasibility*) from the perspectives of participants and delivering clinicians. Recommended triangulation methods to integrate findings from quantitative and qualitative sources will be used to interpret the evaluation findings [35].

## Discussion

This paper describes pre-planned analyses for the HeLP Trial, aiming to reduce risk of data-driven reporting of results. The trial has enrolled the intended sample size and is currently completing follow-up data collection (until December 2020). Any changes from the protocol and analysis plan will be described in the final published report.

## Trial status

We completed participant enrolment on 19 November 2019, and follow-up data collection will conclude in December 2020.

## Supplementary Information

**Additional file 1.**

## Data Availability

De-identified data with accompanying data dictionaries will be made available on reasonable request. All analysis protocols are expected to be made publicly available and published in open access peer reviewed journals. Proposals for data use may be submitted to the principal and corresponding investigator.
