# Supplementary material for: Effectiveness of a Healthy Lifestyle Program (HeLP) for low back pain: statistical analysis plan for a randomised controlled trial
Source: Trials. 2021 Sep 22;22:648. doi: 10.1186/s13063-021-05591-0 (PMC8459477; doi:10.1186/s13063-021-05591-0)
Supplement: Supplementary file 1 — Additional file 1. [file 13063_2021_5591_MOESM1_ESM.docx]

**Additional File 1**

**Table 1: Common medications used for back pain**

| **Timepoint** | **Medication Category** | **HeLP** | **Usual Care** |
| --- | --- | --- | --- |
| Baseline | Medication 1  Medication 2  etc | n/N(%)  n/N(%)  n/N(%) | n/N(%)  n/N(%)  n/N (%) |
| Week 6 | Medication 1  Medication 2  etc | n/N(%)  n/N(%)  n/N(%) | n/N(%)  n/N(%)  n/N(%) |
| Week 12 | Medication 1  Medication 2  etc | n/N(%)  n/N(%)  n/N(%) | n/N(%)  n/N(%)  n/N(%) |
| Week 26 | Medication 1  Medication 2  etc | n/N(%)  n/N(%)  n/N(%) | n/N(%)  n/N(%)  n/N(%) |
| Week 52 | Medication 1  Medication 2  etc | n/N(%)  n/N(%)  n/N(%) | n/N(%)  n/N(%)  n/N(%) |

**Table 2: Descriptions of concomitant healthcare services used for back pain and surgery outcomes.**

| **Timepoint** | **Service Category** | **HeLP** | **Usual Care** |
| --- | --- | --- | --- |
| Baseline | Service 1  Service 2  etc | n/N(%)  n/N(%)  n/N(%) | n/N(%)  n/N(%)  n/N(%) |
| Week 6 | Service 1  Service 2  etc | n/N(%)  n/N(%)  n/N(%) | n/N(%)  n/N(%)  n/N(%) |
| Week 12 | Service 1  Service 2  etc | n/N(%)  n/N(%)  n/N(%) | n/N(%)  n/N(%)  n/N(%) |
| Week 26 | Service 1  Service 2  etc | n/N(%)  n/N(%)  n/N(%) | n/N(%)  n/N(%)  n/N(%) |
| Week 52 | Service 1  Service 2  etc | n/N(%)  n/N(%)  n/N(%) | n/N(%)  n/N(%)  n/N(%) |
| Week 52 | Seen or referred to surgeon (y)  Had back surgery (y)  Believe surgery is appropriate for pain (y) | n/N(%)  n/N(%)  n/N(%) | n/N(%)  n/N(%)  n/N(%) |

**Table 3: Adverse events by group**

| **ICD codes** | **HeLP** | **Usual Care** |
| --- | --- | --- |
| **Week 6** |  |  |
| Adverse Event 1 e.g. XXXX | n/N (%) | n/N (%) |
| Adverse Event 2. e.g. XXXX | n/N (%) | n/N (%) |
| **Week 12** |  |  |
| Adverse Event 1. e.g. XXXX | n/N (%) | n/N (%) |
| Adverse Event 2. e.g. XXXX | n/N (%) | n/N (%) |
| **Week 26** |  |  |
| Adverse Event 1. e.g. XXXX | n/N (%) | n/N (%) |
| Adverse Event 2. e.g. XXXX | n/N (%) | n/N (%) |
| **Week 52** |  |  |
| Adverse Event 1. e.g. XXXX | n/N (%) | n/N (%) |
| Adverse Event 2. e.g. XXXX | n/N (%) | n/N (%) |

**Table 4: Other illnesses requiring medication or health care**

| **Timepoint** | **Illness** | **HeLP** | **Usual Care** |
| --- | --- | --- | --- |
| Baseline | Illness 1  Illness 2  etc | n/N(%)  n/N(%)  n/N(%) | n/N(%)  n/N(%)  n/N(%) |
| Week 6 | Illness 1  Illness 2  etc | n/N(%)  n/N(%)  n/N(%) | n/N(%)  n/N(%)  n/N(%) |
| Week 12 | Illness 1  Illness 2  etc | n/N(%)  n/N(%)  n/N(%) | n/N(%)  n/N(%)  n/N(%) |
| Week 26 | Illness 1  Illness 2  etc | n/N(%)  n/N(%)  n/N(%) | n/N(%)  n/N(%)  n/N(%) |
| Week 52 | Illness 1  Illness 2  etc | n/N(%)  n/N(%)  n/N(%) | n/N(%)  n/N(%)  n/N(%) |

**Table 5: Participant Fidelity, Adherence and participation**

| **Adherence component** | **Intervention** | **Usual Care** |
| --- | --- | --- |
| **Appointment Attendance**   - Initial consultation - Week 3 physiotherapy - Week 3 dietitian - Week 6 physiotherapy - Week 12 physiotherapy - Mean overall | n/N(%)  n/N(%)  n/N(%)  n/N(%)  n/N(%)  N, Mean (SD) | n/N(%)  NA  NA  NA  n/N(%)  N, Mean (SD) |
| **Tame the beast video watched** | n/N(%) | NA |
| **GHS calls completed** | N, Mean (SD) | NA |
| **GHS graduations** | n/N(%) | NA |
| **Referrals to Quitline** | n/N(%) | NA |
| **Calls completed with Quitline** | n/N(%) | NA |

**Table 6: Clinician Fidelity of intervention delivery (Clinician Physiotherapy Checklists)**

| **Outcome** | **Received** |
| --- | --- |
| **Initial** |  |
| 1. 1. Completed physical assessment  - Height and weight measured | n/N (%) |
| 1. 2. Explained HeLP program components 2. 3. Number of consultations 3. 4. Data expectations 4. 5. Outlined HeLP physio and dietitian contact   6. Explained supplemental telephone services | n/N (%)  n/N (%)  n/N (%)  n/N (%) |
| 1. 7. Provided low back pain/neuroscience education | n/N (%) |
| 1. 8. Linked pain to lifestyle behaviours | n/N (%) |
| 1. 9. Outlined GHS and role | n/N (%) |
| 1. 10. Described QL role (if applicable) | n/N (%) |
| 1. 11. Reassurance of GP involvement | n/N (%) |
| 1. 12. Established stage and motivations for change | n/N (%) |
| 1. 13. Acknowledged barriers to change | n/N (%) |
| 1. 14. Goal setting | n/N (%) |
| Appointment length (minutes) | Mean (SD) |
| Total components delivered out of 14 | n/N (%) |
| **Week 3 Physio** |  |
| 1. Recap on HELP program content, education and information from previous session | n/N (%) |
| 1. Update on the Get Healthy Service and encourage and support participation | n/N (%) |
| 1. Review goals and homework | n/N (%) |
| 1. Acknowledge and address barriers to lifestyle change | n/N (%) |
| 1. Mention smoking cessation | n/N (%) |
| 1. Goal setting | n/N (%) |
| Appointment length (minutes) | Mean (SD) |
| Total components delivered out of 6 | n/N (%) |
| **Week 3 Dietitian** |  |
| 1. Recap about HeLP program and importance of diet in lifestyle change for back pain management and chronic disease prevention | n/N (%) |
| 1. Address stage of change and motivations for diet management | n/N (%) |
| 1. Use patient baseline data or brief assessment to help guide conversation and negotiate changes in current diet patient | n/N (%) |
| 1. Negotiate strategies to improve diet | n/N (%) |
| 1. Acknowledge and address barriers to lifestyle change | n/N (%) |
| 1. Update on the Get Healthy Service and encourage and support participation | n/N (%) |
| 1. Goal setting | n/N (%) |
| Appointment length (minutes) | Mean (SD) |
| Total components delivered out of 7 | n/N (%) |
| **Week 6 Physiotherapy** |  |
| 1. Recap on HELP program content, education and information from previous session | n/N (%) |
| 1. Update on the Get Healthy Service and encourage and support participation | n/N (%) |
| 1. Review goals and homework | n/N (%) |
| 1. Acknowledge and address barriers to lifestyle change | n/N (%) |
| 1. Discuss smoking cessation (if applicable) | n/N (%) |
| 1. Goal Setting | n/N (%) |
| Appointment length (minutes) | Mean (SD) |
| Total components delivered out of 6 | n/N (%) |
| **Week 12 physiotherapy** |  |
| 1. Reinforce HeLP principles and ask about patient progress/ experience | n/N (%) |
| 1. Update on the Get Healthy Service; encourage and support participation | n/N (%) |
| 1. Review goals and homework | n/N (%) |
| 1. Acknowledge and address barriers to lifestyle change | n/N (%) |
| 1. Smoking cessation referral (if applicable) | n/N (%) |
| 1. Goal Setting for continued progress/maintenance and long term self-management | n/N (%) |
| Appointment length (minutes) | Mean (SD) |
| Total components delivered out of 6 | n/N (%) |

**Table 7: Common carer or community services support used**

| **Timepoint** | **Task Category** | **HeLP** | **Usual Care** |
| --- | --- | --- | --- |
| Baseline | Task 1  Task 2  etc | n/N(%)  n/N(%)  n/N(%) | n/N(%)  n/N(%)  n/N(%) |
| Week 6 | Task 1  Task 2  etc | n/N(%)  n/N(%)  n/N(%) | n/N(%)  n/N(%)  n/N(%) |
| Week 12 | Task 1  Task 2  etc | n/N(%)  n/N(%)  n/N(%) | n/N(%)  n/N(%)  n/N(%) |
| Week 26 | Task 1  Task 2  etc | n/N(%)  n/N(%)  n/N(%) | n/N(%)  n/N(%)  n/N(%) |
| Week 52 | Task 1  Task 2  etc | n/N(%)  n/N(%)  n/N(%) | n/N(%)  n/N(%)  n/N(%) |

| **Timepoint** | **Service Category** | **HeLP** | **Usual Care** |
| --- | --- | --- | --- |
| Baseline | Service 1  Service 2  etc | n/N(%)  n/N(%)  n/N(%) | n/N(%)  n/N(%)  n/N(%) |
| Week 6 | Service 1  Service 2  etc | n/N(%)  n/N(%)  n/N(%) | n/N(%)  n/N(%)  n/N(%) |
| Week 12 | Service 1  Service 2  etc | n/N(%)  n/N(%)  n/N(%) | n/N(%)  n/N(%)  n/N(%) |
| Week 26 | Service 1  Service 2  etc | n/N(%)  n/N(%)  n/N(%) | n/N(%)  n/N(%)  n/N(%) |
| Week 52 | Service 1  Service 2  etc | n/N(%)  n/N(%)  n/N(%) | n/N(%)  n/N(%)  n/N(%) |

**Table 8: Activities required help with from carers or community support**
